# Supplementary figures and images for: Life history and cancer in birds: clutch size predicts cancer
Source: bioRxiv. 2023 Feb 13:2023.02.11.528100. Preprint. [Version 1] doi: 10.1101/2023.02.11.528100 (PMC9948971; doi:10.1101/2023.02.11.528100)

A

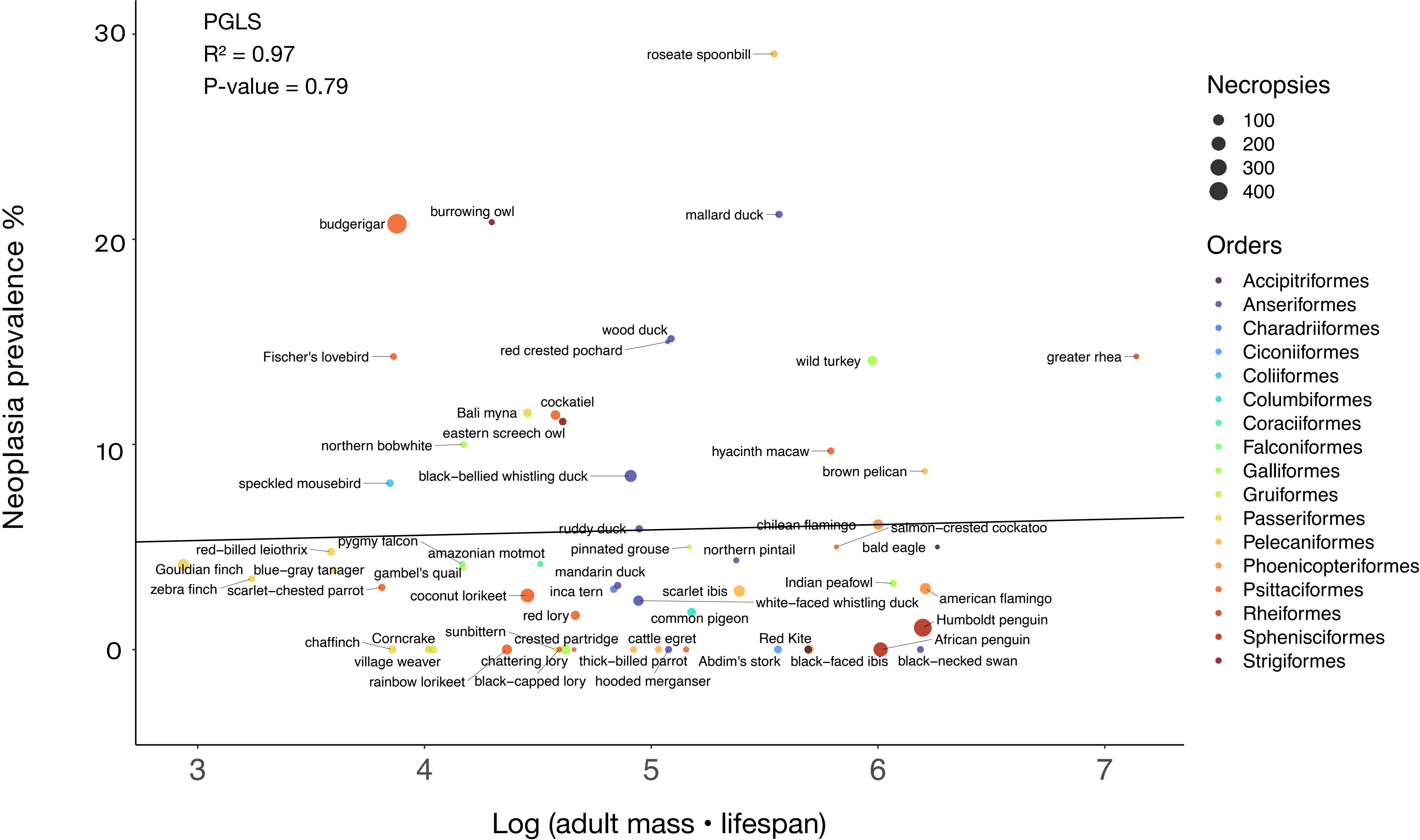

B

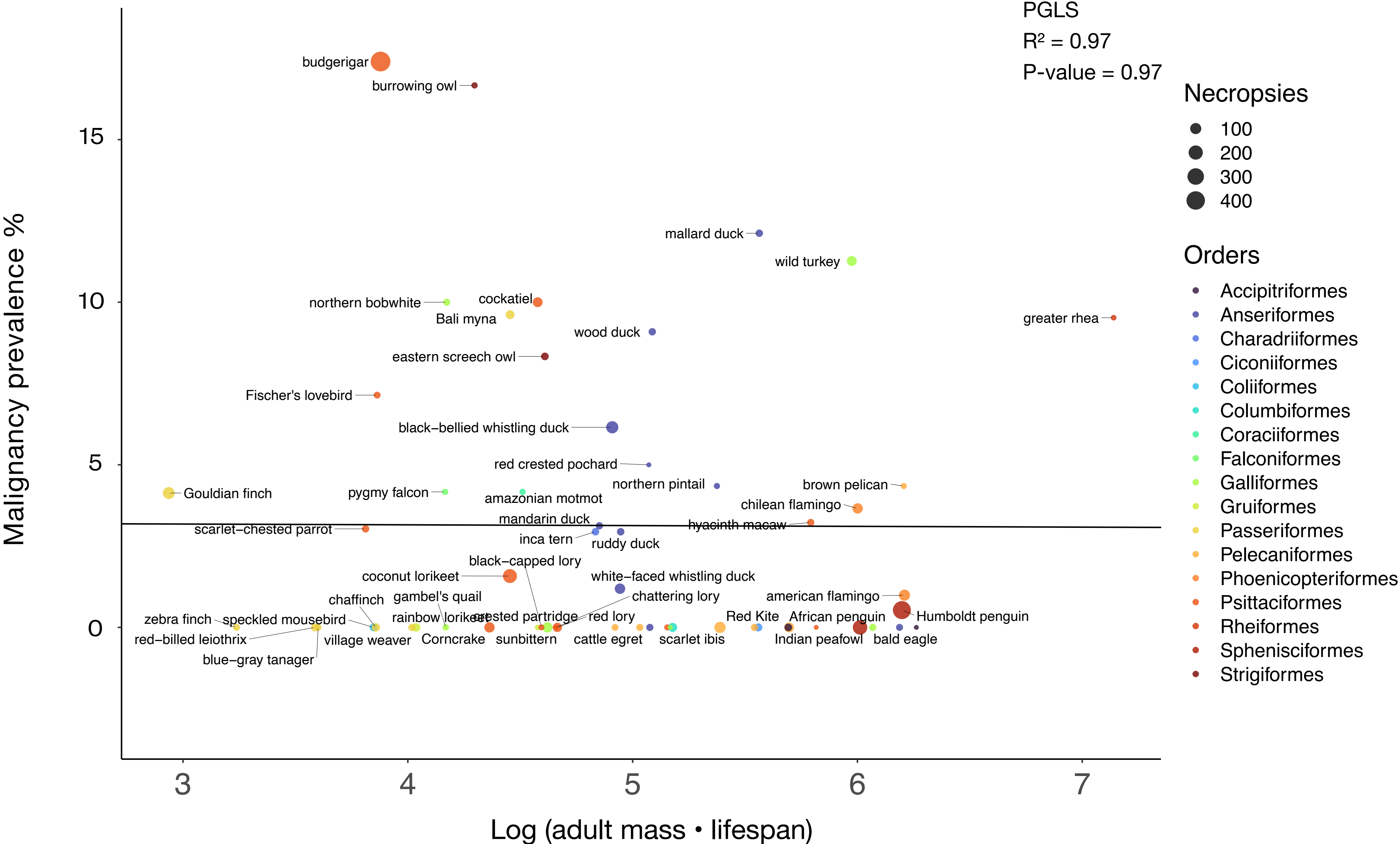

Supplement: Supplement 2 [file media-2.pdf]

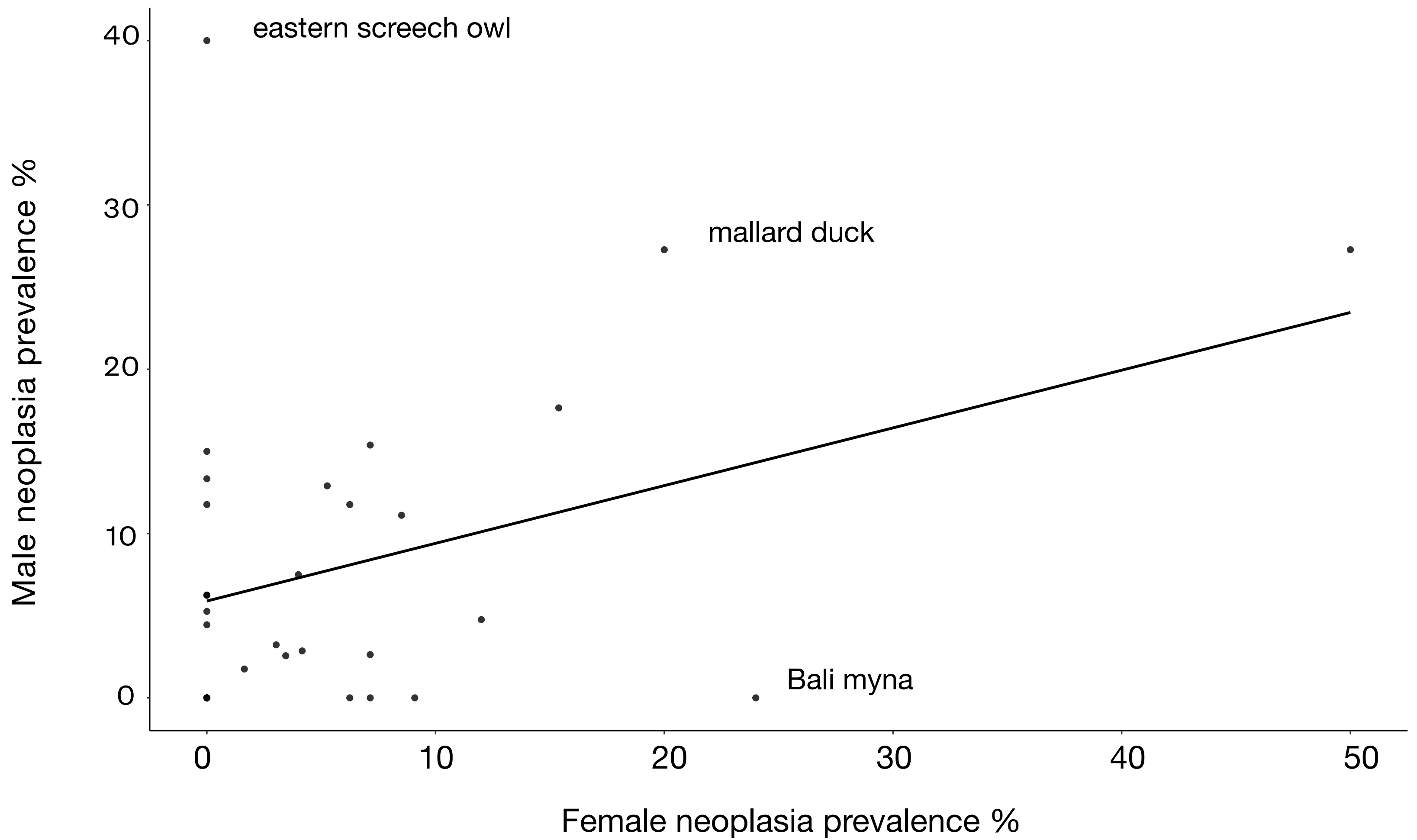

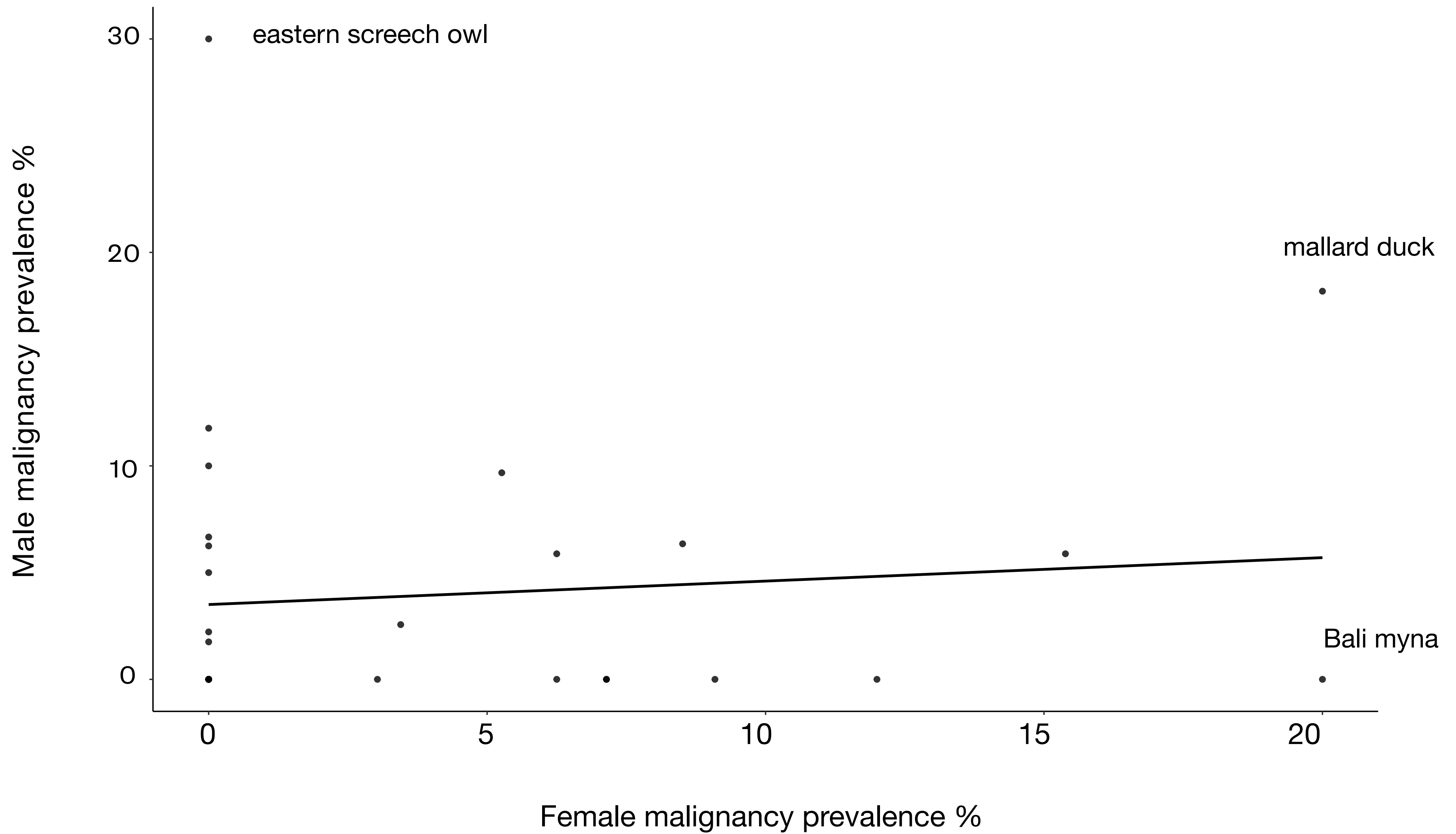

Supplement: Supplement 3 [file media-3.pdf]

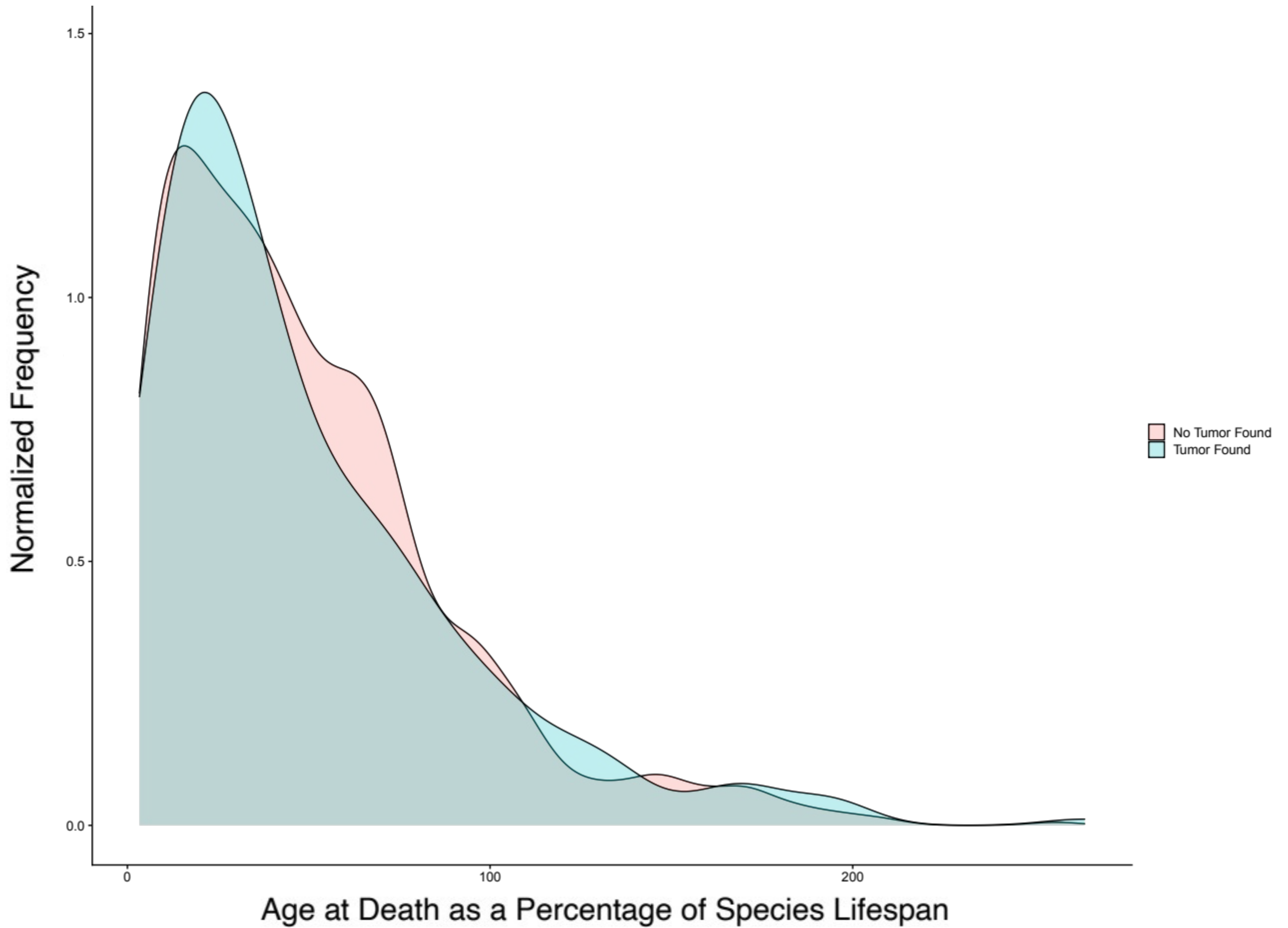

Supplement: Supplement 4 [file media-4.pdf]

A

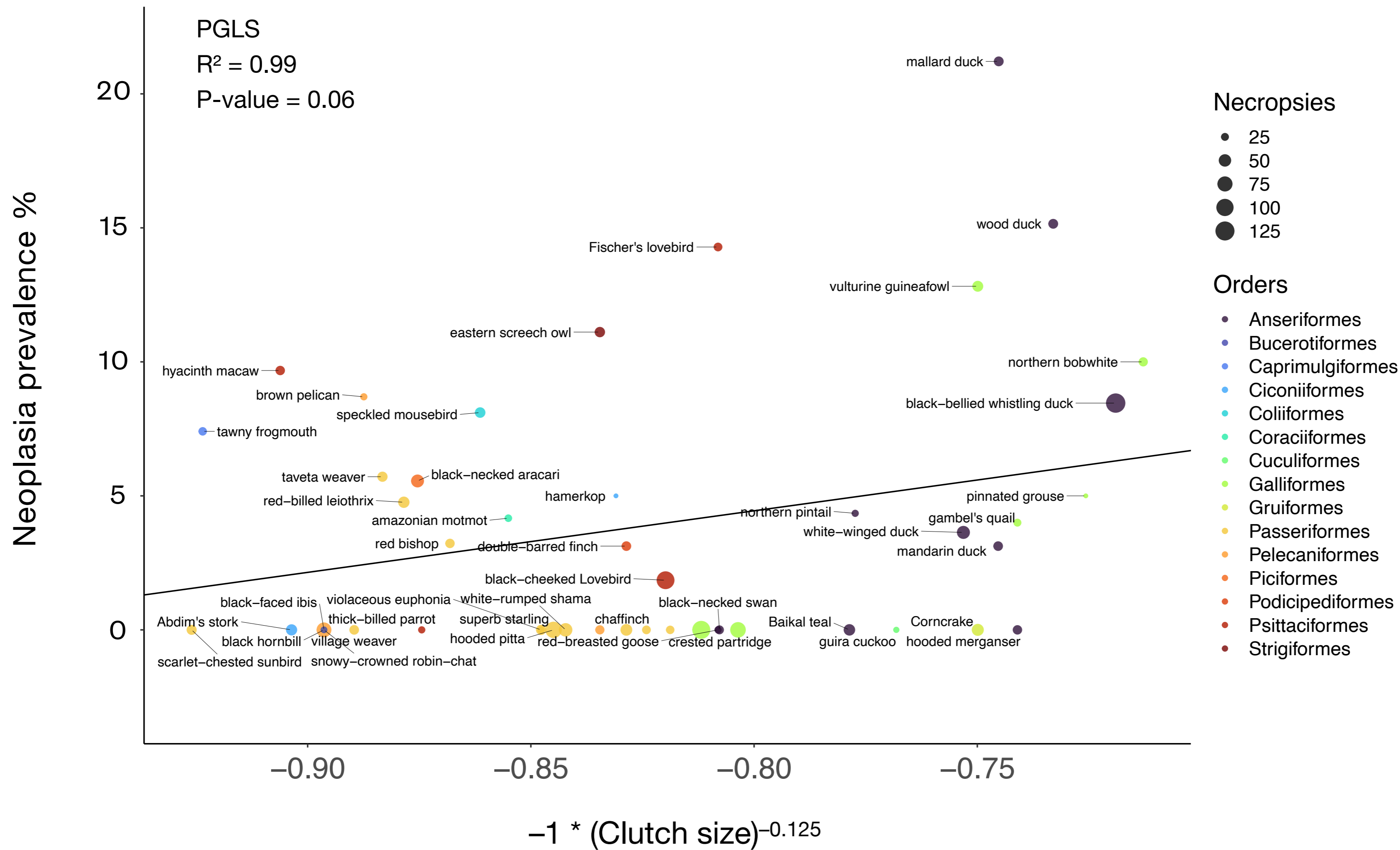

B

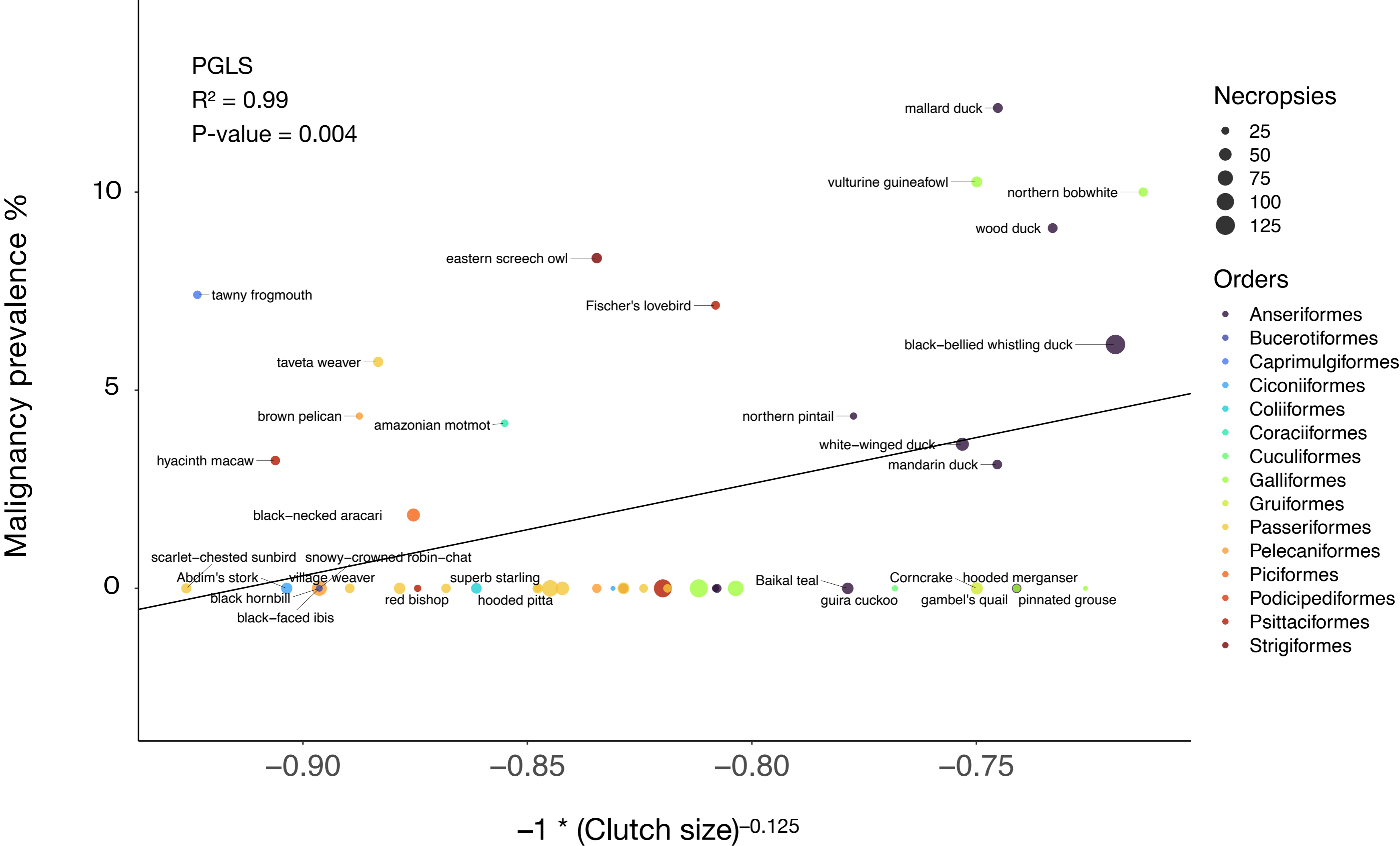

Supplement: Supplement 5 [file media-5.pdf]
