# Supplementary material for: Life history and cancer in birds: clutch size predicts cancer
Source: bioRxiv. 2023 Feb 13:2023.02.11.528100. Preprint. [Version 1] doi: 10.1101/2023.02.11.528100 (PMC9948971; doi:10.1101/2023.02.11.528100)
Supplement: Supplement 6 [file media-6.pdf]

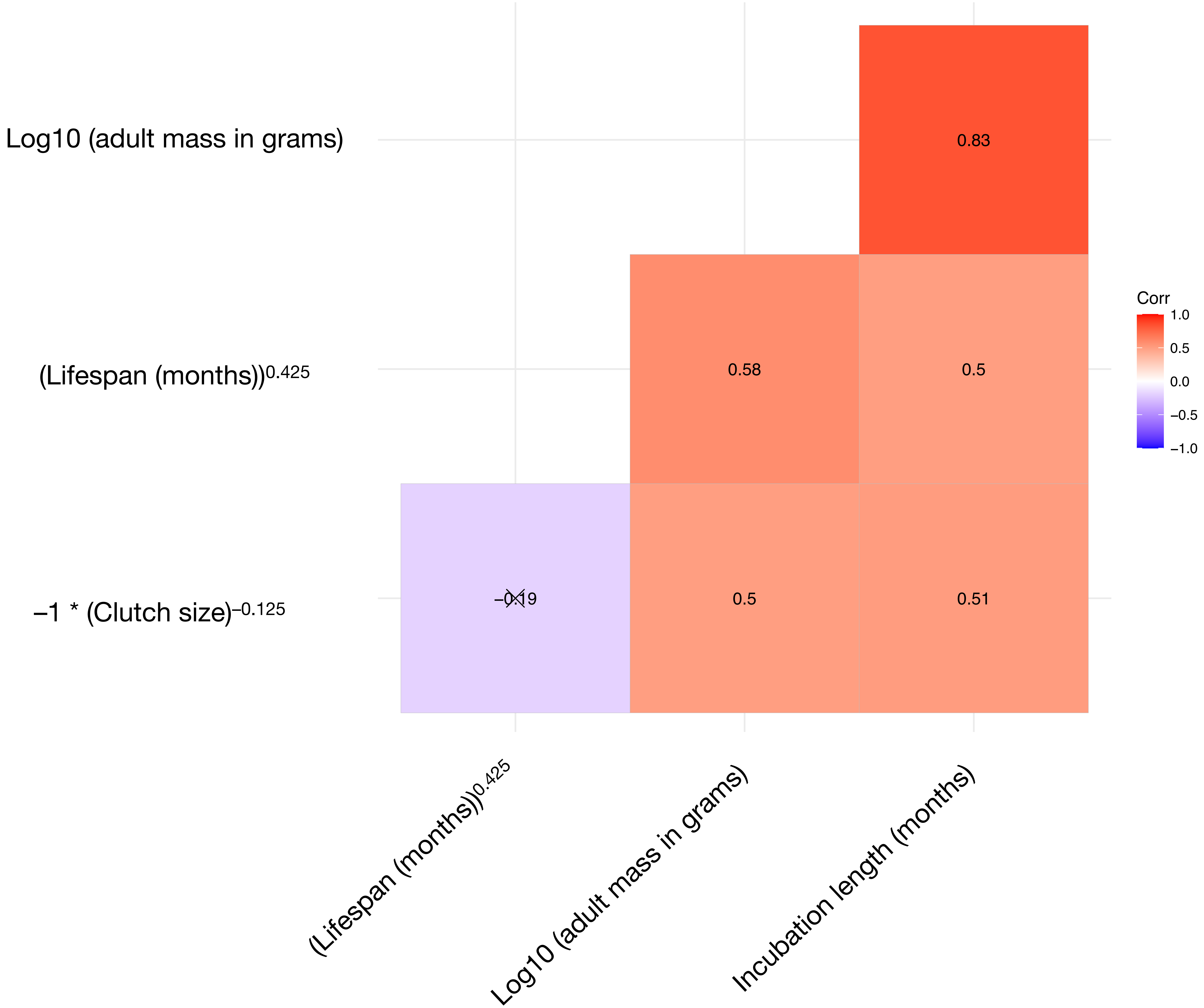

Log10 (adult mass in grams)
(Lifespan (months))^0.425
−1 * (Clutch size)^−0.125
0.83
0.58
0.5
−0.19
0.5
0.51
Corr
1.0
0.5
0.0
−0.5
−1.0
(Lifespan (months))^0.425
Log10 (adult mass in grams)
Incubation length (months)
