# Supplementary material for: Life history and cancer in birds: clutch size predicts cancer
Source: bioRxiv. 2023 Feb 13:2023.02.11.528100. Preprint. [Version 1] doi: 10.1101/2023.02.11.528100 (PMC9948971; doi:10.1101/2023.02.11.528100)
Supplement: Supplement 7 [file NIHPP2023.02.11.528100v1-suuplement-7.pdf]

bioRxiv preprint doi: https://doi.org/10.1101/2023.02.11.528100; this version posted February 13, 2023. The copyright holder for this preprint (which was not certified by peer review) is the author/funder, who has granted bioRxiv a license to display the preprint in perpetuity. It is made available under aCC-BY 4.0 International license.

Supplementary material

Supplementary Figure 1. The $\log_{10}$ of adult mass times lifespan is not correlated with neoplasia prevalence (A) or malignancy prevalence (B) across 57 bird species. The black lines show the phylogenetically-controlled linear regression of the $\log_{10}$ of adult mass times lifespan versus malignancy prevalence or neoplasia prevalence. Adult mass is measured in grams, whereas lifespan is measured in months. Different colors show the different order each species belongs to.

bioRxiv preprint doi: https://doi.org/10.1101/2023.02.11.528100; this version posted February 13, 2023. The copyright holder for this preprint (which was not certified by peer review) is the author/funder, who has granted bioRxiv a license to display the preprint in perpetuity. It is made available under aCC-BY 4.0 International license.

Supplementary Figure 2. No significant sex bias in neoplasia (A) or malignancy prevalence (B) across 31 bird species. Each dot in plot A shows the male neoplasia prevalence and female neoplasia prevalence of a species. Whereas each dot in plot B shows the male malignancy prevalence and female malignancy prevalence of a species.

Supplementary Figure 3. Cancer deaths are not skewed towards old age. Normalized frequency of a species' age at death as a percentage of the species lifespan. Each density plot shows the necropsied individuals that had tumors (blue) and the necropsied individuals that did not have tumors (red). There are 1287 individuals in this distribution from which we have lifespan data.

Supplementary Figure 4. Larger clutch size is correlated with malignancy prevalence (B) but not neoplasia prevalence (A) across 45 bird species after removing domesticated and semi-domesticated species from the analyses . After controlling for species body mass, the positive correlation between clutch size and malignancy prevalence remains significant (*P*-value = 0.004; Table 2). Dot size shows the number of necropsies per species. Colors show the taxonomic order of each species. Black lines indicate the phylogenetically-controlled linear regression of the normalized values of clutch size versus malignancy prevalence or neoplasia prevalence.

Supplementary Figure 5. Pearson's correlation matrix with four life history variables shared by 19 species in our dataset ($\log_{10}$ of adult mass, $\text{lifespan}^{0.425}$, incubation length, –1 ■ $\text{clutch size}^{-0.125}$).

Supplementary data

Life history and cancer dataset used in this study.

Supplementary Table 1. Common species analyzed in Møller et al.[19] and our study. Here we present species' tumor incidence and number of records in Møller et al.[19] versus neoplasia prevalence and number of necropsies in our study. We also present the *P*-values of Fisher's exact test.

**Species (common name)**	**Tumor incidence in Møller et al.'s study (# records)**	**Neoplasia prevalence in this study (# necropsies)**	***P*-value**
Columba livia (rock pigeon)	0% (3)	1.8% (55)	1
Anas acuta (northern pintail)	0% (3)	4.3% (23)	1
Milvus milvus (red kite)	0% (3)	0% (36)	1
Crex crex (corncrake)	0% (4)	0% (47)	1
Fringilla coelebs (chaffinch)	0% (213)	0% (45)	1
Anas platyrhynchos (mallard duck)	4.7% (21)	21.2% (33)	0.13
